# Visualization of the relationship between electrogustometry and whole mouth test using multidimensional scaling

**DOI:** 10.1038/s41598-023-35372-5

**Published:** 2023-05-31

**Authors:** Jong-Gyun Ha, Bo-ra Kim, Ara Cho, Yeonsu Jeong, Min-Seok Rha, Ju-Wan Kang, Hyung-Ju Cho, Joo-Heon Yoon, Chang-Hoon Kim

**Affiliations:** 1 Department of Otorhinolaryngology-Head and Neck Surgery, Chung-Ang University College of Medicine, Gwangmyeong Hospital, Gwangmyeong-Si, Korea; 2 Department of Medicine, Graduate School, Yonsei University, Seoul, Korea; 3 Department of Otorhinolaryngology, Yonsei University College of Medicine, 50-1 Yonsei-Ro, Seodaemun-Gu, Seoul, Korea; 4 Department of Otorhinolaryngology, Gangnam Severance Hospital, Yonsei University College of Medicine, Seoul, Korea; 5 The Airway Mucus Institute, Yonsei University College of Medicine, Seoul, Korea; 6 Korea Mouse Sensory Phenotyping Center, Yonsei University College of Medicine, Seoul, Korea; 7 Global Research Laboratory for Allergic Airway Diseases, Seoul, Korea; 8 Medical Research Center, Yonsei University College of Medicine, Seoul, Korea

**Keywords:** Oral manifestations, Gustatory system

## Abstract

Interpreting the relationship between different taste function tests of different stimuli, such as chemical and electrical stimulation, is still poorly understood. This study aims to analyze visually as well as quantitatively how to interpret the relationship of results between taste function tests using different stimuli. Patients who underwent the whole mouth test and Electrogustometry (EGM) at a tertiary medical center between August 2018 and December 2018 were reviewed retrospectively with electronic medical records. Of the 110 patients, a total of 86 adults who self-reported that their taste function was normal through a questionnaire were enrolled. EGM measured the thresholds of the chorda tympani (CT) and glossopharyngeal nerve (GL) area of the tongue. The whole mouth test measured detection and recognition thresholds for sweet, salty, bitter, sour, and umami taste. Statistical analyses of Pearson’s, Spearman’s rank and polyserial correlation and multidimensional scaling (MDS) was performed. The EGM threshold for the average value of both CT regions and the recognition threshold of the whole mouth test were significantly correlated in sweet, salty, bitter, and sour taste (r = 0.244–0.398,* P* < 0.05), and the detection threshold was correlated only significant in sweet (r = 0.360, *P* = 0.007). In the MDS analysis results, the three-dimensional (*D*) solution was chosen over the 2-*D* solution because of the lower stress. Detection-, recognition threshold of whole mouth test and EGM thresholds of CT and GL area, those were standardized by Z-score, formed well-distinguished sections in the MDS analyses. The EGM threshold of the CT area was closer to the detection and recognition thresholds than the EGM threshold of the GL area. In general, the EGM threshold was closer to the recognition threshold than the detection threshold for each taste. Overall, visualization of the relationship of whole mouth test and EGM by MDS was in good agreement with quantitative analysis. EGM and whole mouth test seem to reflect different aspects of taste. However, when interpreting the EGM results, the EGM threshold of the CT area will show more similarity to the recognition threshold than the detection threshold for the whole mouth test.

## Introduction

Taste, like olfaction, is a complex chemosensory perception that plays an important role in judging the external environment. Until now, various tests have been developed to evaluate taste and have been used in the field of clinical research. Chemical taste tests that evaluate cognition by direct exposure of different concentrations of a chemical to the tongue include the whole mouth test^1^, test strip test^2^, and filtered paper disc method (FPD)^3^. Electrogustometry (EGM) is another test commonly used in clinical settings to check the response to electrical stimulation of the tongue and shows good test–retest reliability^4^. Many studies have been conducted on the relationship between various types of chemical taste function tests and the EGM threshold, but their results remain controversial. It is generally accepted that a single test cannot reflect the global taste function^5^.

Multidimensional scaling (MDS) is a multivariate statistical technique that can graphically represent the similarity (or dissimilarity) of items within a data set^6^. Dimensional reduction is performed by analyzing complex and heterogeneous data through the MDS process. MDS provides a visual representation of data, allowing researchers to understand the relative relationships between entities that are difficult to put into words^7^ MDS was useful for graphical representation of relationships between various chemosensory stimuli^8–11^.

In this study, we intended to visualize the relationship between the detection threshold and recognition threshold of the whole mouth test, one of the most commonly used chemical taste tests, and the EGM threshold using MDS analysis. Qualitative analysis of various tastes using the MDS method has been carried out in the following studies: Metallic and basic tastes^12^, bitter taste substance^13^, and chemesthetic stimuli like spicy substance^14^. However, this study was the first to visualize the similarity or dissimilarity of responses to different taste function tests using MDS.

## Methods

### Ethics approval and consent to participate

This study complies with the Declaration of Helsinki. The Institutional Review Board of the Severance Hospital (Seoul, Korea) approved the study (No. 4–2021-1668), and waived the need for informed consent.

### Subjects

Adult subject (age ≥ 19 years old) who underwent whole mouth test (YSK taste function test kit, Rhico medical, Seoul, Korea) and EGM (Rion TR-06, Sensonics Inc, Haddon Heights, NJ, USA) at a tertiary medical center of Republic of Korea between August 2018 and December 2018 were included in this study. Patients who could not fully follow the examiner's instructions due to cognitive dysfunction such as Alzheimer's disease or Parkinson's disease were excluded. Of the 110 subjects, 24 were excluded because they answered that their taste had deteriorated in the questionnaire. Finally, 86 subjects were included in this study.

### Whole mouth test and EGM

We employed a commercially developed and widely used chemical taste function test for assessing taste function in our study. This test has been cited as a reference in various scientific articles, demonstrating its reliability and validity^15–17^. The test consists of a set of solutions for the five basic tastes: sweet (sucrose), salty (sodium chloride), bitter (quinine hydrochloride), sour (citric acid), and umami (monosodium glutamate). Each taste is provided at six different concentrations, represented by a scale ranging from 1 (lowest concentration) to 6 (highest concentration). Each taste solution consisted of six concentrations of dilutions (Table 1)^18^. Patients’ taste function was measured through both detection and recognition threshold levels with a rating scale ranging from 1 (lowest concentration) to 7 (no response to the highest concentration test stimuli). Lower threshold level means better taste function. The dropping of taste solutions starts with the lowest concentration for each of the five tastes in a random order and proceeds until the subject get the correct answers twice in a row. If the subject answered wrong taste, administration of taste solution was performed at one level higher. The detection threshold was defined as the lowest concentration of the test solution that the subject could consistently recognize the chemical stimuli on their tongue regardless of taste, and the recognition threshold was defined as the lowest concentration that could be answered by distinguishing each taste.

**Table 1:** Threshold level of taste stimuli for the whole mouth test.

Taste	Material	Threshold level (concentration, mg/ml)						
		1	2	3	4	5	6	7
Sweet	Sucrose	4.8	9.7	19.5	39	78.1	156.3	No response
Salty	Sodium chloride	0.6	1.2	2.4	4.8	9.6	19.2	
Bitter	Quinine	0.05	0.1	0.2	0.4	0.8	1.6	
Sour	Citric acid	0.2425	0.485	0.97	1.95	3.91	7.81	
Umami	Monosodium glutamate	2.0	4.0	8.0	16.0	32.0	64.0	

EGM was used to measure electrical taste thresholds of both the chorda tympani nerve (CT) area (anterior tongue) and glossopharyngeal nerve (GL) area (posterior tongue) of both sides of the tongue. The electrical stimulation sites were chosen based on their anatomical locations: the CT area site was 1.5 cm apart from the tip of the tongue^19^, while the GL area site was close to the foliate papillae^20^. The tongue's taste bud is electrically stimulated for 0.5–2 s by a metal probe with a diameter of 5 mm within the stimulation range of -6 dB (4 μA) to 34 dB (400 μA). Steps of 2 dB were used during measurement^21^. Subjects do not know the stimulation time and intensity of the current, and when the subjects feel taste or similar sensation in the tongue, they press the button to inform that they have recognized the electrical sensation. The threshold is the level at which the subject accurately felt twice, and there should be no response at the level below the level. If there was no response to the maximum intensity stimulus (34 dB), the threshold was treated as 36 dB^22^.

### Statistical analysis

The association between detection-, recognition-, and EGM threshold was calculated via Pearson’s correlation, Spearman’s rank correlation and polyserial correlation. Non metric MDS with the alternating least squares scaling (ALSCAL) algorithm was performed for visualization of the relationship between whole mouth test and EGM^23^. MDS analysis was performed after adjusting and standardizing the results of each variable using Z-scores to adjust for different rating scales of each detection and recognition threshold and EGM threshold. Dissimilarity was expressed via relative distance (Euclidean distance) from each variable and graphically showed in the 2-D and 3-D dimension. The distance between two variables in the MDS map tends to be inversely proportional to the Pearson correlation coefficient between the two variables. Variables that were close to each other had similar characteristics, and variables that were far apart were judged to have large differences. An indicator that measures how well the proximity (similarity or non-similarity) between variables entered in MDS is converted to the distance on the finally derived coordinate space is called fit measures, and Kruskal’s stress and R-quared correlation (RSQ) are used for fit measures. Between the MDS results expressed in low dimensions, the dimension with lower stress and higher RSQ was selected as a suitable model because it had less distortion. SPSS 25.0 (IBM Corp., Armonk, NY, USA) and R, version 4.2.2.2 (R Foundation for Statistical Computing) was used for the statistical analysis.

## Results

### Patients and clinical data

Of the 86 subjects, 27 were women. Mean age of the subject was 46.3 (SD 17.6) years. Table 2 showed the summary of the results of the whole mouth test and EGM threshold.

**Table 2:** The result of threshold for the whole mouth test and the EGM (n = 86).

		DT	RT	*rho*†	*P*-value
		Mean (SD)	Mean (SD)		
Whole mouth test (threshold, level)	Sweet	2.13 (1.07)	3.12 (1.42)	0.588	< 0.001
	Salty	3.01 (1.14)	4.22 (1.34)	0.586	< 0.001
	Bitter	2.57 (1.18)	3.71 (1.09)	0.232	0.032
	Sour	2.14 (0.98)	4.14 (1.62)	0.352	0.001
	Umami	1.78 (1.02)	3.27 (1.66)	0.473	< 0.001
	Mean	2.33 (0.75)	3.69 (0.81)	0.487	< 0.001

		CT	GL	*r*‡	*P*-value
		Mean (SD)	Mean (SD)		
EGM (threshold, dB)	Right	2.54 (9.83)	17.30 (12.51)	0.688	< 0.001
	Left	1.58 (9.21)	16.19 (12.94)	0.638	< 0.001
	Mean	2.01 (9.40)	16.74 (12.57)	0.674	< 0.001

*EGM* electrogustometry, *DT* detection threshold, *RT* recognition threshold, *SD* standard deviation, *r* correlation coefficients, *dB* decibel, *CT* chorda tympani nerve, *GL* glossopharyngeal nerve.

^†^Spearman’s rank correlation.

^‡^Pearson’s correlation.

### Correlation between detection and recognition threshold of whole mouth test

Overall, the detection threshold for each taste was lower than its corresponding recognition threshold. The detection and recognition thresholds for sweet (*rho* = 0.588), salty (*rho* = 0.586), bitter (*rho* = 0.232), sour (*rho* = 0.352), and umami (*rho* = 0.499) tastes showed significant correlation (*P* < 0.05, respectively). There was also a significant correlation between the mean values of the detection and recognition threshold levels for all five tastes (*rho* = 0.487, *P* < 0.001) (Table 2).

### Correlation between EGM thresholds for both CT and GL nerve areas

The EGM threshold of the CT area was generally lower than that of the GL area. Each EGM threshold for both CT and GL nerve areas showed a significant correlation regarding laterality and mean values (*r* = 0.638 ~ 0.688, *P* < 0.001, respectively) (Table 2).

### Correlation between EGM threshold and the detection or recognition threshold

Table 3 depicts the correlation between the mean value of the EGM threshold for both tongue sides and the detection or recognition thresholds. The mean value of EGM threshold of both tongue sides in CT area showed significant correlation with the recognition threshold of sweet (*r* = 0.244, *P* = 0.018), salty (*r* = 0.287, *P* = 0.007), bitter (*r* = 0.398, *P* = 0.001), sour taste (*r* = 0.271, *P* = 0.010) and their mean values (*r* = 0.405, *P* < 0.001) except umami (*P* = 0.714). For the detection threshold, unlike recognition threshold, only sweet taste (*r* = 0.360, *P* = 0.001) and the mean value of all tastes (*r* = 0.252, *P* = 0.020) showed significant correlation with the CT area EGM threshold. GL area EGM threshold showed a significant correlation with recognition threshold of salty (*r* = 0.304, *P* = 0.006), bitter taste (*r* = 0.284, *P* = 0.005) and mean value of 5 tested tastes (*r* = 0.288, *P* = 0.001) and also showed a significant correlation with detection threshold of salty taste (*r* = 0.223, *P* = 0.035).

**Table 3:** Correlation of detection and recognition threshold level and EGM threshold.

EGM	Whole mouth test											
	Sweet		Salty		Bitter		Sour		Umami		Mean	
	DT	RT	DT	RT	DT	RT	DT	RT	DT	RT	DT	RT
CT^a^	0.360**	0.244*	0.167	0.287*	0.042	0.398**	0.197	0.271*	0.168	0.065	0.252*	0.405**
GL^b^	0.224	0.164	0.223*	0.304**	− 0.109	0.284**	0.027	0.138	0.138	0.012	0.123	0.288**

*EGM* electrogustometry, *DT* detection threshold, *RT* recognition threshold, *CT* chorda tympani nerve, *GL* glossopharyngeal nerve.

^a^Mean value of right and left EGM thresholds of the chorda tympani area.

^b^Mean value of right and left EGM thresholds of the glossopharyngeal nerve area.

Statistical analysis: polyserial correlation.

*P < 0.05.

**P < 0.01.

***P < 0.001.

### Qualitative visualization of whole mouth test and EGM using MDS

**Figure 1:**
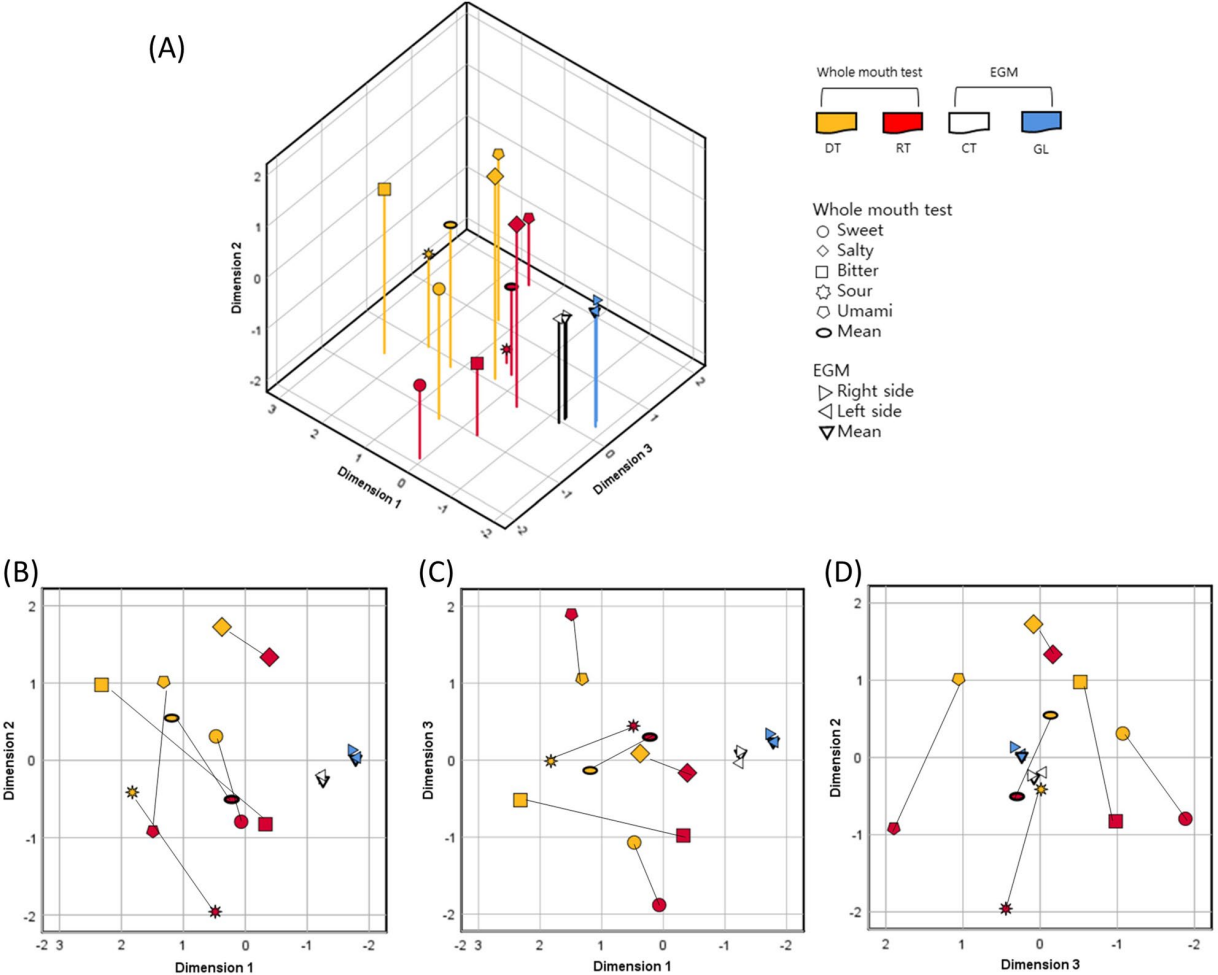
(**A**) *3-D* configurations of MDS for both the whole mouth test and EGM using the Euclidean distance model. Detection and recognition thresholds and EGM thresholds, standardized by Z-score, formed well-distinguished areas. (**B**) Dimension 1 and 2, (**C**) Dimension 1 and 3, (**D**) Dimension 2 and 3. Black line connects detection and recognition threshold of each taste. D, dimension; MDS, multidimensional scaling; EGM, electrogustometry; DT, threshold of detection; RT, threshold of recognition; CT, chorda tympani nerve; GL, glossopharyngeal nerve.

Figure 1A is a qualitative visualization of the relationship between the detection and recognition thresholds for each taste and the EGM thresholds of the CT and the GL nerve areas. The Euclidean distance between each stimulus and the coordinates calculated therefrom are included in Supplement 1. Because of the lower Kruskal’s stress value, a *3-D* solution (stress 0.096) was chosen instead of a *2-D* solution (stress 0.165). The RSQ also improved when calculating the 3-D solution (0.938) compared to the 2-D solution (0.853) (Table 4). The results of EGM and whole mouth test were well distinguished from each other based on the MDS analysis. The EGM threshold of the GL nerve area was relatively far from detection and recognition thresholds of the whole mouth test versus that of the CT nerve area. The recognition threshold for each taste was generally closer to the EGM threshold of the CT area than the detection threshold. Mean value of detection or recognition threshold for the basic tastes located at the center of each. To clarify, Fig. 1B–D are presented as flat graphs, displaying only two of the three axes from the original three-dimensional graph.

**Table 4:** *2-D* and *3-D* configurations for multidimensional scaling.

	*2-D* configuration		*3-D* configuration	
	Kruskal’s stress	*RSQ*	Kruskal’s stress	*RSQ*
Value	0.165	0.853	0.096	0.938

*D* dimensional, *RSQ* R-squared correlation.

## Discussion

Overall, the MDS analysis of this study visually showed that detection or recognition thresholds of the whole mouth test and EGM reflect different aspects of taste. However, there was some significant correlation among these different taste function tests. In particular, the results of whole mouth test were closer to EGM threshold of the CT nerve area, which is considered to have the most contribution among the various nerves involved in taste. Further, EGM threshold showed more similarity to recognition threshold than to detection threshold. This qualitative MDS analysis was in good agreement with the quantitative analysis of the Pearson correlation.

The use of MDS in our study offers an intuitive way to visualize the relationships between the different taste function tests without relying on explicit words and numerical values. It allows for a better understanding and interpretation of complex relationships in the data, which may be particularly useful when dealing with a variety of sensations that are ambiguous to describe.

In the MDS mapping, the relationship between each variable is visually structured and displayed, independent of the researcher's intention. In this study's MDS mapping, each taste test has a clearly distinct area from one another along the dimension 1 axis. The dimension 1 axis may represent the different characteristics of detection and recognition thresholds of the whole mouth test and EGM thresholds of the CT and GL areas. Interestingly, the relative distances between detection and recognition thresholds of the same taste seem to be generally closer compared to the detection or recognition thresholds of other tastes. For instance, the distance between detection and recognition thresholds of salty taste appears to be closer than the detection or recognition thresholds of other tastes.

The mean values of detection and recognition thresholds of the whole mouth test and the mean values of EGM thresholds of the CT or GL area are situated close to the 0 value of the dimension 3 axis. Moreover, detection and recognition thresholds of the five basic tastes are generally represented by dimensions 2 and 3. In Fig. 1D, the connections between detection and recognition thresholds of each taste appear to be relatively vertical, indicating that the differences in dimension 3 values of detection and recognition thresholds of the same taste are small. Dimension 3 may represent the characteristics of each taste, while dimension 2 may illustrate different characteristics of detection and recognition thresholds.

The results of our study demonstrate that the EGM threshold of the CT area is more strongly correlated with the whole mouth test than the GL area. This finding is supported by both qualitative analyses using MDS and quantitative analysis. One potential explanation for this observed correlation may be attributed to the anatomical features of the tongue. The CT nerve is responsible for innervating the anterior two-thirds of the tongue, which contains a higher density of taste buds compared to the posterior one-third, where the GL nerve is located. This anatomical difference in taste bud distribution could be the underlying reason for the stronger correlation observed between the whole mouth test and the EGM threshold of the CT area.

The use of EGM for assessment of taste function has had some history of controversy in that the taste perception with the stimulus is unfamiliar, so further investigation in its utility is important. Several studies compared EGM with chemical taste tests. Sweet, salty, and sour taste excluding bitterness of CT nerve area through FPD had a significant correlation with EGM threshold in a study^21^. Another study reported a significant negative correlation between the total taste strip score and the EGM threshold^24^. On the other hand, only salty taste was significantly correlated with EGM threshold in another study comparing EGM and whole mouth test, whereas sweet, sour, and bitter taste were not^25^. However, it should be interpreted in consideration of that the subjects of this study were not only patients with simple loss of taste, but also 28.2% of patients underwent radiotherapy for head and neck cancer. In the present study, EGM threshold of the CT nerve area was significantly correlated with the recognition threshold for sweet, salty, sour, and bitter tastes, except for umami. Comprehensively judging the results of previous studies, it seems that there is some correlation between the EGM and chemical taste tests, despite evaluating responses to different stimuli. However, the result for each individual taste was inconsistent between each study. These inconsistent results may be due to the low correlation between different chemical taste function tests^5,26^. Even detection and recognition thresholds of whole mouth test are considered partially independent phenotypes^27^. Since EGM is a method of measuring the response to nerve conduction rather than directly measuring chemical taste, caution is needed in the interpretation of its results^21^. Although the paragraphs above dealt with studies reporting correlations between EGM thresholds and the chemical taste test thresholds, interesting differences between EGM and chemical taste tests have often been reported. One study reported that EGM was better at detecting age-related decline in taste than taste strip test^28^. Another study found that EGM were more sensitive to deviations of taste and showed no signs of recovery in patients with taste impairment after middle ear surgery unlike taste strip test^29^. In the MDS analysis in this study, chemical taste test and EGM constituted a well-distinguished area. Unlike chemical taste tests, EGM is difficult to use to measure specific taste characteristics, but EGM appears to clearly reflect one aspect of taste^30^.

A variety of taste function tests have been developed so far, and many studies have been conducted on the clinical utility of each. In a study dealing with the sensitivity and specificity of EGM and chemical taste test for poor tasters based on a visual analogue scale (VAS) score of 50%, both tests tended to show low sensitivity and high specificity, making it difficult to use as a screening test for taste disorders^31^. However, it should be considered that the self-assessment of taste like VAS has low reliability^32^. Both EGM and chemical taste function tests are still generally accepted as important diagnostic tools in the diagnosis of taste deterioration^33^. A comprehensive interpretation of these various taste test results is required in the clinical field. MDS would be helpful in this aspect because it can be performed without relying on explicit words and it is especially useful when dealing with a variety of sensations that are ambiguous to describe^34^.

There are some limitations for this study. First, there may be criticisms on the subject of this study itself, which is comparison of the response between taste function tests using different stimuli. However, how to interpret EGM compared to chemical taste tests is a long-standing research topic. Through this study, we tried to break away from the quantitative analysis and intuitively interpret the relationship through unintentional visualization. Moreover, the results of this qualitative analysis were broadly consistent with those of the quantitative analysis. Second, this study was designed as a retrospective study, so there might be selection bias compared to a well-controlled prospective study. Therefore, it is necessary to confirm the results shown in this study through follow-up prospective studies. In addition to the comparison of EGM and whole mouth test, visualization of the relationship between other taste function tests such as taste strip test or FPD would be an interesting research topic.

## Conclusion

Overall, judging by the MDS analysis, it is clear that whole mouth test and EGM are taste function tests that evaluate different aspects of electric and chemical taste. These two tests are statistically significant in some, but have weak correlations. The EGM threshold of the CT area may show more similarity to the recognition threshold than that of the GL area. This study may help clinicians intuitively interpret EGM and whole mouth test results, and provide additional insight into the complex relationships between different taste function tests.

## Supplementary Information

Supplementary Information.

## Data Availability

The data sets used and analyzed in this study are available from the corresponding author upon reasonable request.
